# Analysis of patient information leaflets (PILs), used in clinical trials using the Informed Consent Evaluation instrument (ICEi)

**DOI:** 10.1186/1745-6215-12-S1-A121

**Published:** 2011-12-13

**Authors:** Katie Gillies, Wan Huang, Zoe Skea, Seonaidh Cotton

**Affiliations:** 1 Health Services Research Unit, University of Aberdeen, Aberdeen, AB25 2ZB, UK; 2 Academic Urology Unit, University of Aberdeen, Aberdeen, AB25 2ZB, UK

## Background

Informed consent (IC) is regarded as a cornerstone of ethical healthcare research and is a requirement for most clinical research studies [1]. Guidelines suggest that prospective clinical trial participants should understand a basic amount of information about trials in order to provide valid IC. However, poor participant understanding of the research processes, a lack of knowledge about the expectations and demands of trials and insufficient support when faced with the decision has been demonstrated across a range of clinical areas [2,3]. As such, the existing approach to obtaining IC for clinical trials is not optimal. Therefore, it is important to investigate the effectiveness of the current consent process to examine how well existing PILs conform to empirically developed standards for promoting high quality decisions. We propose that the process could be improved by drawing on existing research in the fields of decision making and decision support interventions.

## Methods

Websites of Clinical Trial Units registered with the UK Clinical Research Collaboration were screened to identify open access PILs from ongoing or recently completed randomised controlled trials (RCTs). A total of 60 PILs were identified and 20 were purposively sampled, for analysis using the Informed Consent Evaluation instrument (ICEi). The ICEi was developed by combining informed consent guidelines and the International Patient Decision Aid Standards (IPDAS), which describe detailed recommendations about the content and delivery of information to facilitate high quality decisions for treatment or screening. Two independent raters scored PILs according to the items presented in the ICEi, any disagreement was resolved by a third rater.

## Results

Variation existed amongst the PILs in terms of overall scores and scores for specific items. Some aspects were consistently poor across all PILs analysed, namely: presenting probabilities; clarifying and expressing values; and structured guidance in deliberation and communication.

## Conclusions

PILs could be improved to facilitate better quality decision making with regard to participation in an RCT. Patients need to be better supported to make more informed decisions about their clinical trial participation, which are in line with their personal values and preferences. Better informed decisions about participation may result in patients being retained throughout the duration of the trial, as their decisions will be linked to more realistic expectations and be more in line with their personal values and preferences.
